# Sox2 Is Required for Maintenance and Differentiation of Bronchiolar Clara, Ciliated, and Goblet Cells

**DOI:** 10.1371/journal.pone.0008248

**Published:** 2009-12-14

**Authors:** David H. Tompkins, Valérie Besnard, Alexander W. Lange, Susan E. Wert, Angela R. Keiser, April N. Smith, Richard Lang, Jeffrey A. Whitsett

**Affiliations:** Division of Pulmonary Biology in the Perinatal Institute and Division of Pediatric Ophthalmology, Cincinnati Children's Hospital Medical Center and the University of Cincinnati College of Medicine, Cincinnati, Ohio, United States of America; KU Leuven, Belgium

## Abstract

The bronchioles of the murine lung are lined by a simple columnar epithelium composed of ciliated, Clara, and goblet cells that together mediate barrier function, mucociliary clearance and innate host defense, vital for pulmonary homeostasis. In the present work, we demonstrate that expression of Sox2 in Clara cells is required for the differentiation of ciliated, Clara, and goblet cells that line the bronchioles of the postnatal lung. The gene was selectively deleted in Clara cells utilizing *Scgb1a1-Cre*, causing the progressive loss of *Sox2* in the bronchioles during perinatal and postnatal development. The rate of bronchiolar cell proliferation was decreased and associated with the formation of an undifferentiated, cuboidal-squamous epithelium lacking the expression of markers of Clara cells (Scgb1a1), ciliated cells (FoxJ1 and α-tubulin), and goblet cells (Spdef and Muc5AC). By adulthood, bronchiolar cell numbers were decreased and Sox2 was absent in extensive regions of the bronchiolar epithelium, at which time residual Sox2 expression was primarily restricted to selective niches of CGRP staining neuroepithelial cells. Allergen-induced goblet cell differentiation and mucus production was absent in the respiratory epithelium lacking *Sox2*. *In vitro*, Sox2 activated promoter-luciferase reporter constructs for differentiation markers characteristic of Clara, ciliated, and goblet cells, *Scgb1a1*, *FoxJ1*, and *Agr2*, respectively. Sox2 physically interacted with Smad3 and inhibited TGF-β1/Smad3-mediated transcriptional activity *in vitro*, a pathway that negatively regulates proliferation. *Sox2* is required for proliferation and differentiation of Clara cells that serve as the progenitor cells from which Clara, ciliated, and goblet cells are derived.

## Introduction

The respiratory epithelium is lined by a diversity of distinct epithelial cell types that vary in abundance along its proximal-distal axis. Larger cartilaginous airways (e.g. trachea and bronchi) are lined by a pseudostratified epithelium consisting primarily of basal, ciliated, non-ciliated secretory cells (Clara cells), and goblet cells. Non-cartilaginous airways are lined primarily by a simple columnar epithelium consisting of Clara and ciliated cells. Together, the respiratory epithelium of conducting airways plays a critical role in pulmonary homeostasis, maintaining barrier function and sterility. The lung is continuously exposed to particles, toxicants, and microbial pathogens that are generally cleared from the airways by an extensive defense system, mediated in part by mucociliary clearance, secretion of fluids, and antimicrobial molecules, as well as by the activities of the innate and acquired host defense systems. Cellular composition of conducting airways varies during development, and is highly responsive to acute and chronic injury in the postnatal lung. Differences in cell differentiation of the conducting airways as compared to the peripheral alveolar regions of the lung are established early in fetal development [1], [2]. The diverse cell types lining the trachea, bronchi, bronchioles, and alveoli become highly differentiated in the perinatal and postnatal period, during which the respiratory epithelium is proliferative. While cell turnover and proliferation is relatively low in the mature lung, the respiratory epithelium is capable of extensive proliferation following injury [3], [4], [5], [6]. Basal and Clara cells are known to serve as progenitor cells in the conducting airways, while type II epithelial cells proliferate during repair of the alveoli [3], [4], [7]. After severe bronchiolar injury, toxicant resistant Clara cells and bronchoalveolar stem cells (BASCs) residing in distinct cellular niches, also have been proposed as relatively specialized stem cells that play a role in repair and/or carcinogenesis [7], [8]. Cellular hyperplasia and metaplasia of the airway epithelium are associated with common, chronic lung disorders, including those induced by smoking, chronic obstructive pulmonary disease (COPD), asthma, cystic fibrosis, lung cancer, and interstitial lung diseases. While goblet cells are not highly abundant in the normal airway, goblet cell numbers and mucus hyperproduction are commonly associated with both acute and chronic infection, allergy, and exposure to toxicants (for example, cigarette smoke) [9], [10], [11], [12]. Proliferation and differentiation of the diverse cells lining the respiratory tract are influenced by complex signaling and transcriptional programs that are active during formation of the respiratory epithelium and during its repair. The roles of a number of transcription factors, including members of the NKX, FOX, TCF/LEF, SOX, ETS, RAR, p53/p63, Krupple-like factors, the Notch signaling pathway, and others play a role in formation and differentiation of the respiratory epithelium [13], [14], [15]. In postnatal lung, Clara cells are capable of self-renewal and differentiate into both ciliated cells and goblet cells, and there is evidence that the Clara cell serves as a common progenitor of the various cell types lining the peripheral conducting airways [16], [17], [18].

The transcription factor Sox2, a member of the SRY-high mobility box transcription factor family, is expressed in epithelial cells of the foregut, including pharynx, esophagus, trachea, bronchi, and bronchioles, but is excluded from the peripheral and alveolar regions of the lung [19]. Sox2 has been implicated in the control of differentiation in several contexts, including maintenance of self-renewal and pluripotency in embryonic and neural retinal stem cells, differentiation of tongue taste buds and ear sensory cells from progenitors, and production of pluripotent stem cells [20], [21], [22], [23], [24]. Sox2 is also expressed in developing respiratory epithelium and is restricted to conducting airways of the mature lung [25]. Sox2 is induced in the bronchiolar epithelium during repair after toxicant induced injury [26]. Germline deletion of *Sox2* results in blastomere death [27]. Reduction of Sox2 using hypomorphic alleles caused hearing loss, defects in eye formation, and failure of tracheal/esophageal separation [21], [22], [24], [28]. Overexpression of *Sox2* in lung epithelium during early development disrupted branching morphogenesis, causing cystic lungs and neonatal death [25]. Deletion of *Sox2* from primitive foregut under control of the *Nkx2.5* promoter caused malformations of the larynx and trachea, an abnormal tracheal epithelium, and neonatal death [19]. While these data suggest a role for Sox2 in regulating embryonic differentiation in multiple tissues, the requirement for Sox2 in bronchiolar cells of the postnatal lung is unknown.

To determine the role of *Sox2* in differentiation and maintenance of the respiratory epithelium after birth, we deleted *Sox2* from mouse Clara cells beginning in late gestation. During the first four postnatal weeks, when the mouse lung undergoes dramatic growth, we observed progressive loss of columnar Clara, ciliated, and goblet cells, resulting in an airway lined by a mixture of cuboidal and squamous epithelial cells lacking morphological and biochemical markers of normal differentiated airway. In adult mice, *Sox2* was required for the differentiation of goblet cells and production of mucus following allergen exposure *in vivo*, and activated promoter-luciferase reporters for the differentiation markers *Scgb1a1*, *FoxJ1*, and *Agr2 in vitro*. Sox2 loss resulted in reduced proliferation *in vivo*, and antagonized TGF-β1 signaling by interacting with Smad3 *in vitro*. In spite of the loss of differentiated ciliated, Clara, and goblet cells, mice in which *Sox2* was extensively deleted in the respiratory epithelium survived normally under vivarium conditions. Sox2 is required for the maintenance and differentiation of Clara, ciliated and goblet cells after birth.

## Results

### Localization of Sox2 in the Respiratory Epithelium

**Figure 1:**
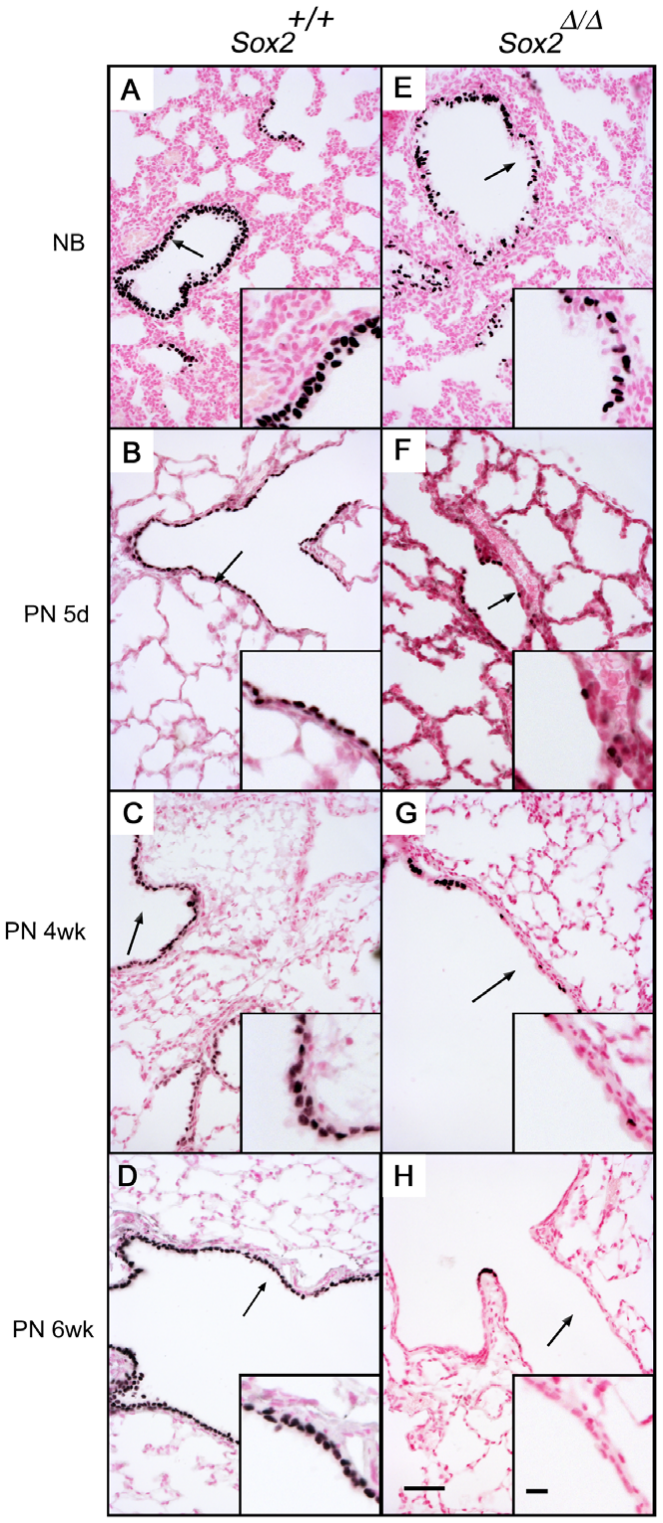
Progressive deletion of *Sox2* from the bronchiolar epithelium. In control mice, strong nuclear staining for Sox2 is observed in all epithelial cells lining the conducting airways (A–D). *Sox2* was progressively deleted from the conducting airway epithelium of *Sox2^Δ/Δ^* mice (E–H) from late gestation to adulthood. Arrows indicate regions shown in insets. Scale bar is 50 µm, inset scale bar is 10 µm for all panels. NB, newborn; PN, postnatal.

Immunohistochemical analysis was used to identify the sites of Sox2 expression in the developing lung epithelium. A rabbit antiserum was produced against a synthetic mouse Sox2 peptide (aa 111 to 140). Sites of expression in the mouse embryo were consistent with previously described sites of *Sox2* mRNA. In newborn, postnatal, and adult mice (post-natal day 0.5, 5, 28, and 42) strong nuclear staining for Sox2 was observed in all cells of the conducting airway epithelium (Fig. 1A–D). Sox2 was absent from the alveolar epithelium at all time points. Sox2 staining was observed in the developing brain, pharynx, esophagus, and intestinal tract (not shown), consistent with its known pattern of expression provided by the GenePaint mRNA database [29].

### Progressive Deletion of Sox2 from Lung Epithelium

Cre-recombinase was expressed under control of the rat *Scgb1a1* promoter [30] to delete *Sox2* from the conducting airway epithelium. Previous studies demonstrated the activity of this promoter in Clara cells in the conducting airway epithelium from E16-17 and after birth [31], [32]. *Scgb1a1-Cre^tg/-^/Sox2^flox/flox^ (Sox2^Δ/Δ^)* mice were produced in which *Sox2* was selectively and permanently deleted from Clara cells. Since both basal and Clara cells serve as progenitor cells in the proximal or “cartilaginous” airways, the present studies were focused primarily to the role of Sox2 in the bronchiolar regions that are lined by a simple columnar epithelium consisting of Clara, ciliated, and a relatively smaller number of neuroepithelial cells. To assess the effectiveness of Cre-mediated deletion, Sox2 staining was performed on lung sections from newborn to 6 week old mice. In newborn *Sox2^Δ/Δ^* mice, the numbers of Sox2 stained cells were decreased; however, both Sox2-positive and Sox2-negative cells were detected. In control littermates, all bronchiolar cells were Sox2-positive (Fig. 1A,E). By postnatal day 5, *Sox2* deletion was more widespread and varied among mice. In all mice, bronchiolar regions wherein Sox2 was absent lost their columnar characteristics, becoming cuboidal-to-squamous in shape (Fig. 1B,F). At 4 and 6 weeks after birth, Sox2-positive cells were occasionally observed in some lobes (not shown). In lobes with nearly complete loss of Sox2, infrequent small clusters of Sox2 positive cells were present (Fig. 1H). Serial lung sections stained for Sox2 and CGRP demonstrated that these Sox2-positive clusters were neuroendocrine cells, a cell type in which the *Scgb1a1* promoter is inactive [1]. Taken together, the progressive loss of *Sox2* in the conducting airways is consistent with the sites of expression of Cre recombinase directed by the rat *Scgb1a1* promoter [1], [30], [31], [32].

### Conditional Deletion of Sox2 Results in Cuboidal-to-Squamous Epithelium Lacking Goblet, Clara, and Ciliated Cells in the Conducting Airway Epithelium

**Figure 2:**
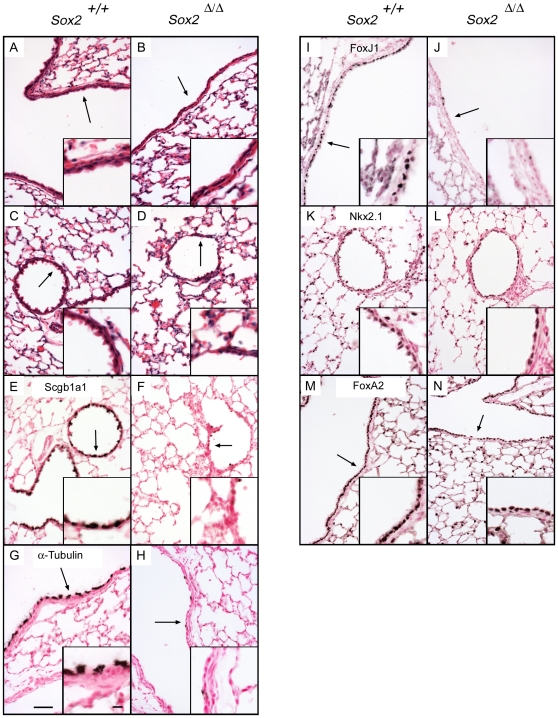
Lack of ciliated and Clara cell selective markers after deletion of *Sox2*. In four week old mice, hematoxylin and eosin staining of control and *Sox2^Δ/Δ^* lung sections demonstrated abnormal low cuboidal-to-squamous epithelium in large bronchioles (A, B) and small bronchioles (C,D). Immunohistochemical staining demonstrated loss of CCSP (Scgb1a1) a Clara cell marker (E,F), ciliated cell markers α-tubulin and FoxJ1 (G–J). Lung epithelial markers Nkx2.1 and FoxA2 were unchanged in control vs *Sox2^Δ/Δ^* mice (K–N). Arrows indicate regions shown in insets. Scale bar is 50 µm, inset scale bar is 10 µm.

**Figure 3:**
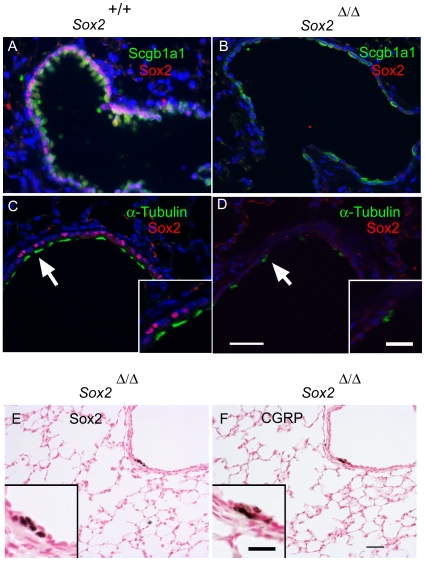
Maintenance of Sox2 expression in neuroepithelial cells. Immunofluorescence staining of six week old *Sox2^Δ/Δ^* mice demonstrated Sox2-positive ciliated and Clara cells in control mice (A,C), the extensive deletion of Sox2, and the presence of rare ciliated and Clara cells lacking Sox2 in *Sox2^Δ/Δ^* mice (B,D). Serial sections from *Sox2^Δ/Δ^* mice (E,F) identified a cluster of more cuboidal, untargeted cells which stained for Sox2 (E) and a distinct subset of Sox2 reactive cells expressing the neuroendocrine cell marker CGRP in which the *Scgb1a1* promoter is not active (F). Arrows indicate regions shown in insets. Scale bars: 20 µm (A–D), 40 µm (E,F). Inset scale bars: 5 µm (A–D), 10 µm (E,F).

The conducting airway epithelium of *Sox2* ^Δ/Δ^ mice was lined with a single layer of low cuboidal and squamous cells that lacked the “domed” morphology characteristic of Clara cells, the apical cilia characteristic of ciliated cells, or the goblet shape of mucus producing cells (Fig. 2A–D). In adult lung, immunohistochemical staining for Scgb1a1 (a Clara cell marker, Fig. 2E,F), FoxJ1 and acetylated α-tubulin (ciliated cell markers, Fig. 2G–J), and Alcian blue staining for mucins (see below) demonstrated the absence of Clara, ciliated, and goblet cells at most sites of *Sox2* deletion. The Clara cell marker, CCSP (Scgb1a1), was lost at most sites of Sox2 deletion at birth and thereafter (Fig. 2E,F). Immunostaining for the lung epithelial markers TTF-1 (thyroid transcription factor 1) (Nkx2.1) and FoxA2 was not affected in *Sox2^Δ/Δ^* airways (Fig. 2K–N). Keratin-14 and p63 were not detected in the bronchiolar epithelium of controls or in *Sox2^Δ/Δ^* mice (data not shown). Co-staining of *Sox2^Δ/Δ^* lung sections for Sox2/Scgb1a1 and Sox2/α-tubulin demonstrated that the majority of cells lacking Sox2 also lacked Scgb1a1 and α-tubulin. The number of ciliated cells expressing α-tubulin decreased with age. By six weeks of age, few ciliated cells remained in the bronchioles of the *Sox2* ^Δ/Δ^ mouse. Of the remaining few, co-staining for Sox2 and α-tubulin demonstrated that most were Sox2-negative (Fig. 3D). In spite of extensive loss of normal conducting airway epithelial cells, pulmonary inflammation was not observed in *Sox2^Δ/Δ^* mice, and the mice have survived normally for more than 6 months in the vivarium.

### Sox2 Is Required for Differentiation of Goblet Cells Following Allergen Exposure

**Figure 4:**
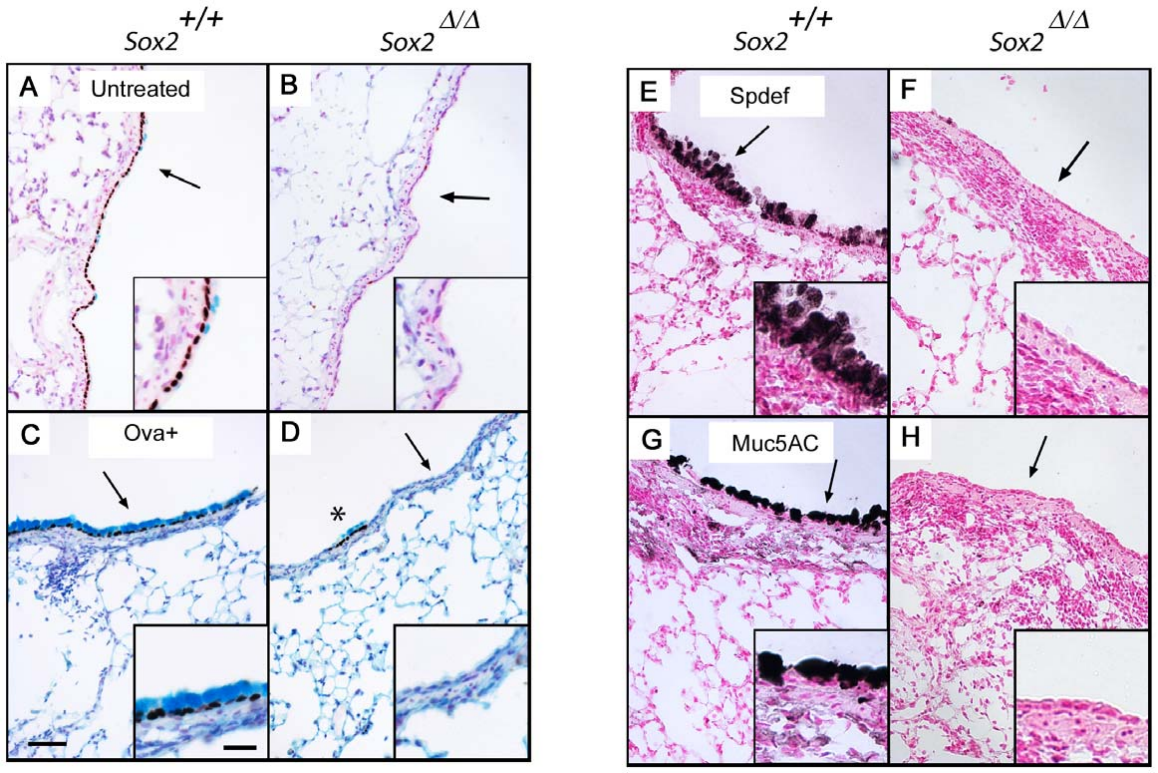
Sox2 is required for goblet cell differentiation after pulmonary allergen sensitization. Lung sections from normal four week old mice were co-stained for mucins with Alcian blue and Sox2 demonstrating the presence of goblet cells in the airways of control mice (A) but not in *Sox2^Δ/Δ^* littermates (B). Response to allergen challenge (C–H): In control mice, Alcian blue reactive cells expressed Sox2 (C,D), and the goblet cell associated proteins Spdef (E,F) and Muc5AC (G,H). Extensive goblet cell differentiation was observed in bronchi and larger bronchioles of control (C,E,G) but not *Sox2^Δ/Δ^* mice (D,F,H), in spite of the robust inflammatory response seen in both groups. Asterisk in D indicates a small cluster of Sox2-positive (untargeted) goblet cells. Arrows indicate regions shown in insets. Scale bar is 50 µm, inset scale bar is 10 µm. OVA+, ovalbumin treatment.

Infrequent goblet cells were observed in some large bronchioles of four week old control mice, but were not observed in *Sox2^Δ/Δ^* littermates, suggesting that Sox2 is required for differentiation of goblet cells or their precursors after birth (Fig. 4A,B). To test whether goblet cell differentiation was inducible by allergen exposure of *Sox2^Δ/Δ^* mice, 6 week old *Sox2^flox/flox^* control and *Sox2^Δ/Δ^* mice were sensitized to ovalbumin by intraperitoneal injection. After three weeks of sensitization, mice received ovalbumin by nasal aspiration and lungs were harvested when the mice were 10 weeks old. Lung sections were immunostained for Sox2 and co-stained with Alcian blue to identify mucin-rich goblet cells. In control animals, dramatic goblet cell metaplasia, with strong staining for Muc5AC and nuclear Sox2 and Spdef was observed in bronchi and larger bronchioles (Fig. 4C,E,G). In contrast, goblet cells were absent in *Sox2* ^Δ/Δ^ bronchiolar epithelium. Neither Spdef (an Ets-like factor required for goblet cell differentiation) nor Muc5AC (a pulmonary mucin) were induced (Fig. 4F,H). Rare clusters of Sox2-positive goblet cells were observed in some large airways of *Sox2^Δ/Δ^* lungs, where they were associated with incomplete *Sox2* deletion (asterisk in Fig. 4D), while inflammation was evident in both control and *Sox2* ^Δ/Δ^ mice. These results demonstrate that Sox2 is required for goblet cell differentiation during allergen challenge.

### Reduced Proliferation and Decrease in Cell Density in the Bronchioles of Sox2^Δ/Δ^ Mice

**Figure 5:**
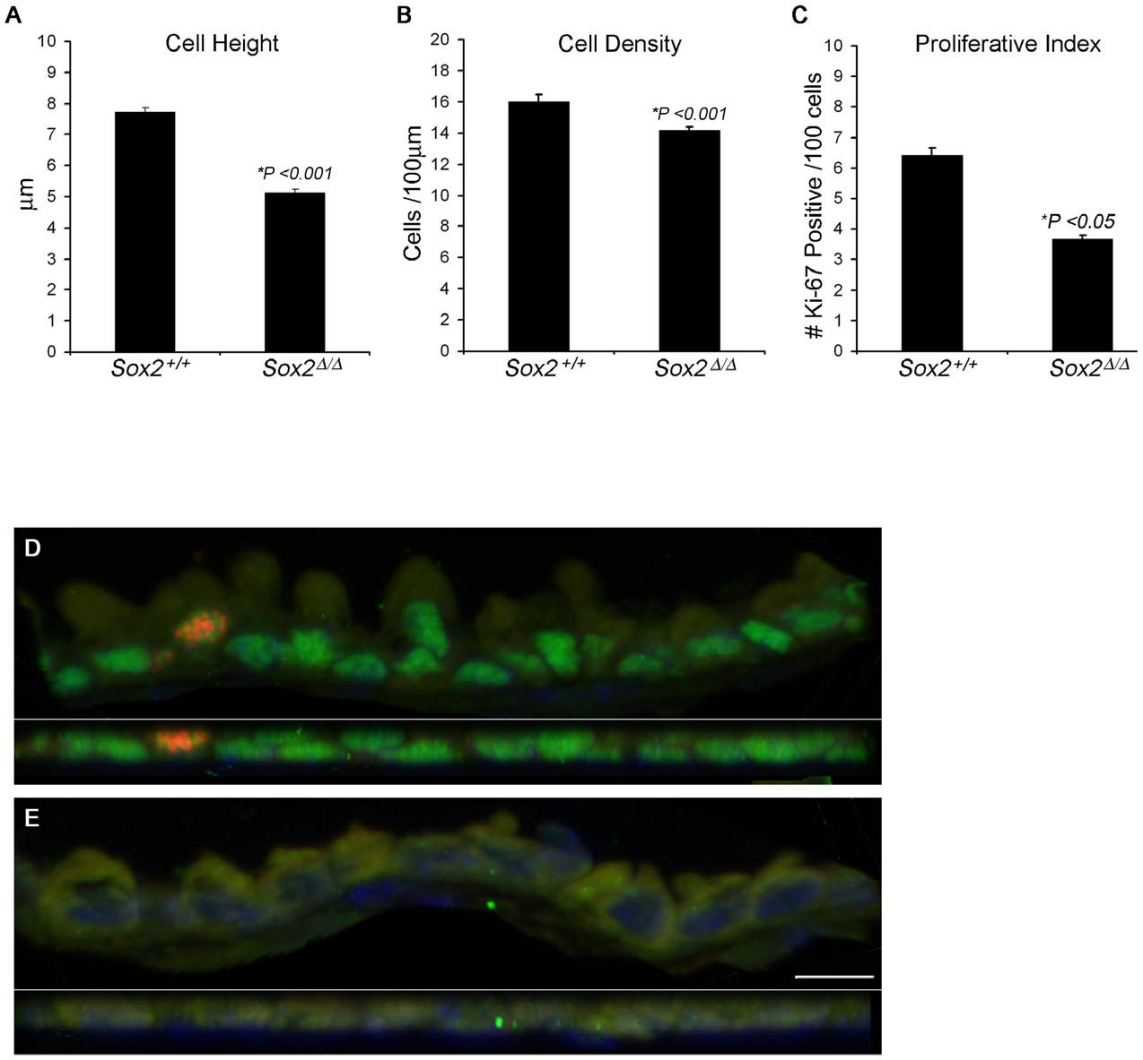
Decreased proliferation and decreased cell density after deletion of *Sox2*. Bronchiolar cells were co-stained for Sox2 (green) and Ki-67 (red). Average cell height, cell density, and proliferation index was assessed by morphometric analysis (A,B,C). Representative bronchiolar epithelium of control (D) and *Sox2^Δ/Δ^* (E) are shown in side view (upper portion) and plan view (lower portion). Graphs depict mean±standard error of the mean.

Murine lung undergoes rapid growth in the first four weeks of life. At 4 weeks of age, immunohistochemical staining for the proliferation marker Ki-67 demonstrated a significant decrease (42%) in the proliferative index in *Sox2* ^Δ/Δ^ mice. To assess whether cell shape was changed, morphometric analysis was performed. Cell width increased 13%, cell height decreased 33%, and cell density decreased 12% compared to controls (p<.001), consistent with the shift from columnar to the cuboidal-squamous morphology seen histologically (Fig. 5). *Sox2* deletion did not result in increased apoptosis, as measured by immunohistochemical staining for cleaved Caspase 3 (data not shown).

### Sox2 Activates Promoter-Luciferase Constructs for Scgb1a1, FoxJ1, and Agr2

**Figure 6:**
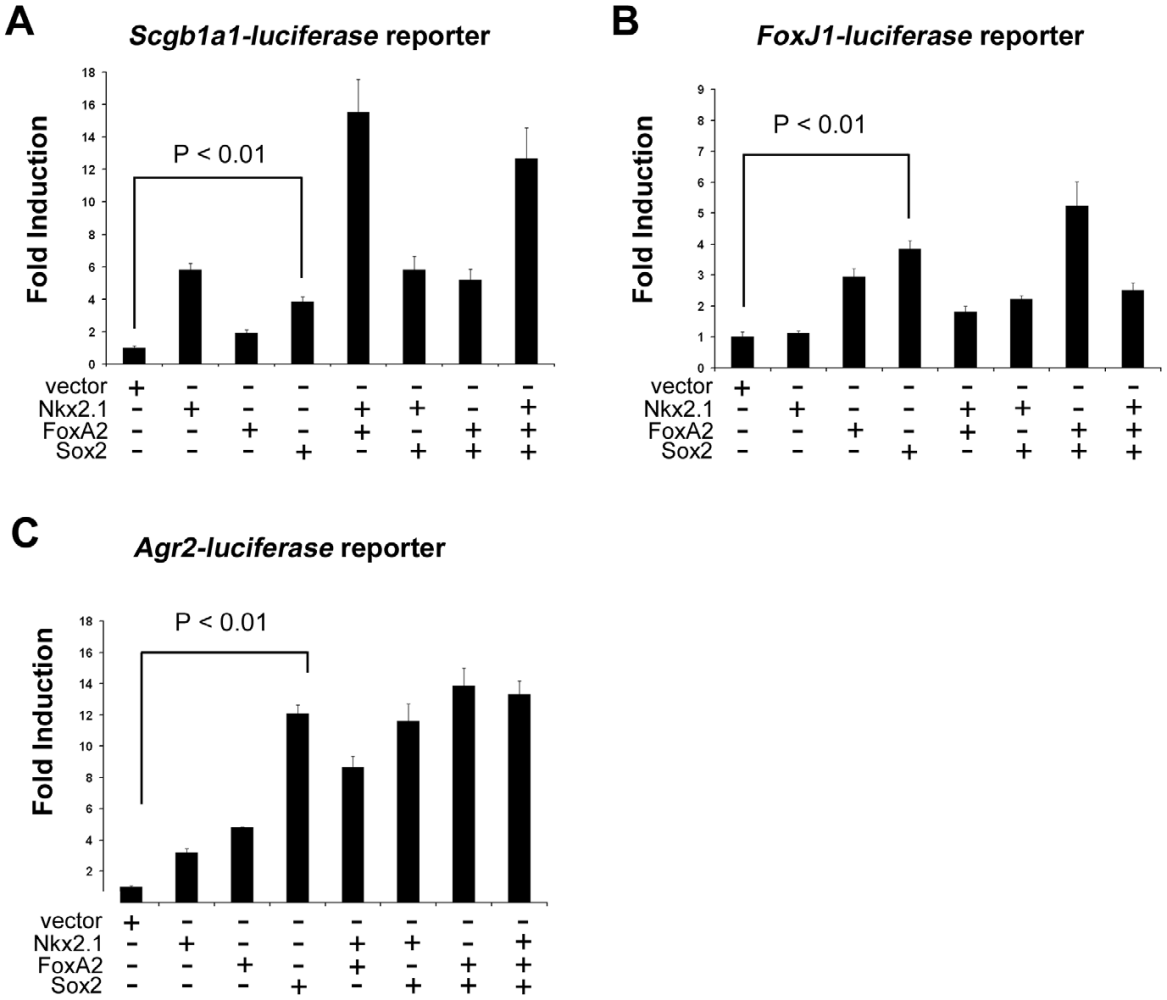
Sox2 induced promoter activity for differentiation markers *Scgb1a1*, *FoxJ1*, and *Agr2*. HBECs were transfected with (A) Scgb1a1-luciferase reporter (0.5 µg), (B) FoxJ1-luciferase reporter (0.5 µg), or (C) Agr2-luciferase reporter (0.5 µg) in the presence of various combinations of Sox2 (0.1 µg), Nkx2.1 (0.1 µg), and FoxA2 (0.1 µg). Sox2 induced modest activation of *Scgb1a1*, *FoxJ1*, and *Agr2* (A,B,C). Synergistic activation of *Scgb1a1* by Nkx2.1 and FoxA2 was observed (A). Sox2 did not synergize with Nkx2.1 or FoxA2 to regulated *Scgb1a1*, *FoxJ1*, or *Agr2* constructs. Graphs depict representative results of 3 separate experiments performed in triplicate, and are expressed as the mean±standard deviation.

Since the deletion of the *Sox2* gene resulted in a loss of differentiation, we sought to identify a mechanism by which Sox2 influenced differentiation. Nkx2.1 and FoxA2 are known regulators of respiratory epithelial differentiation markers, and normal expression levels of these transcription factors were observed in *Sox2^Δ/Δ^* mice. To test the hypothesis that Sox2 is required for Nkx2.1 or FoxA2 activity, promoter-luciferase reporter assays were performed using Sox2 alone and Sox2 plus Nkx2.1 or FoxA2. Sox2 alone induced activation of the *Scgb1a1*, *FoxJ1*, and *Agr2* promoters (Fig. 6A,B,C), markers of the Clara cell, ciliated cell, and goblet cell, respectively. While synergistic activation of the *Scgb1a1* promoter by Nkx2.1 and FoxA2 was observed (Fig. 6A), combination of Sox2 with Nkx2.1 or FoxA2 resulted in additive but not synergistic activation (Fig. 6A,B,C). Sox2, Nkx2.1, and FoxA2 (alone or in combination) did not activate a *Spdef-luciferase* construct (data not shown). Taken together, these data demonstrate that Sox2 is a positive regulator of selected markers of differentiation, but not a synergistic partner of Nkx2.1 or FoxA2.

### Sox2 Interacts with Smad3 and Inhibits TGF-β1/Smad3 Signaling

**Figure 7:**
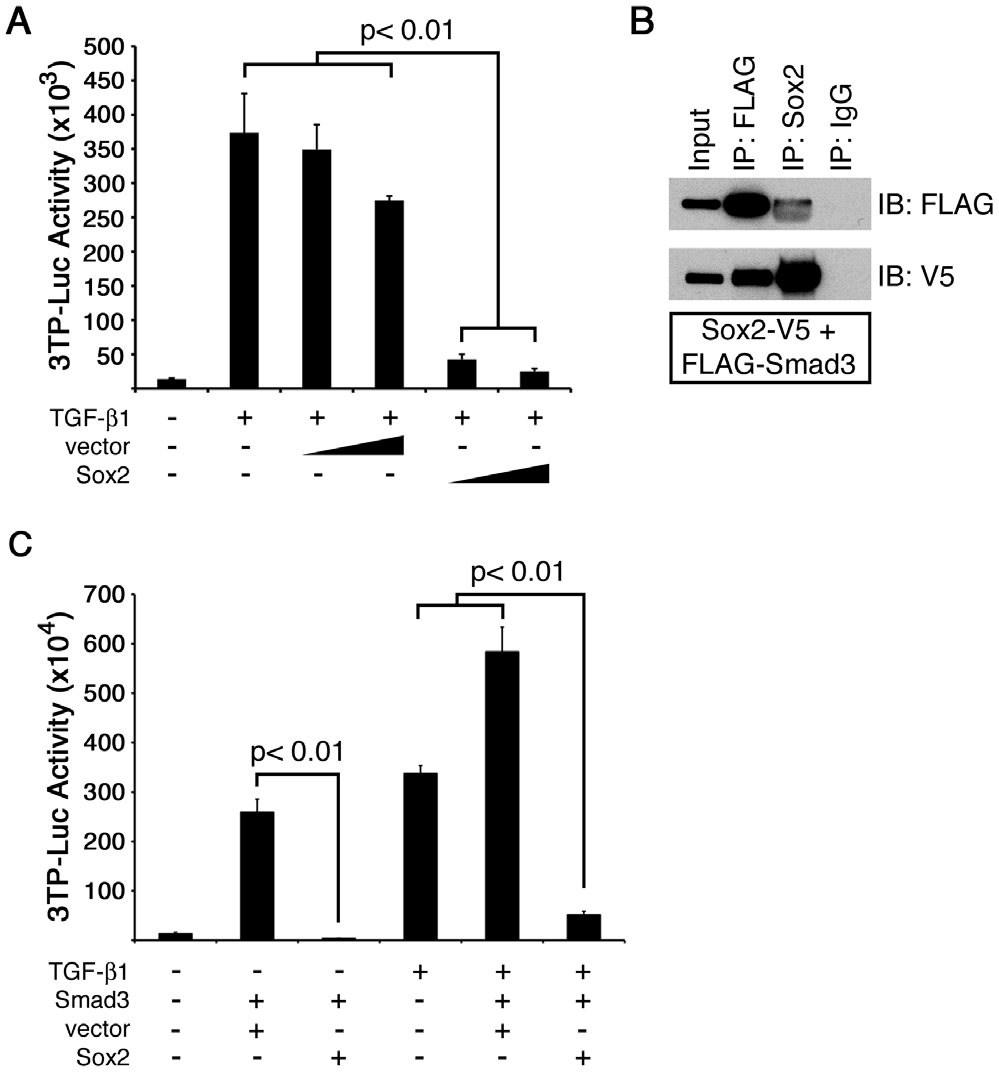
Sox2 inhibited TGF-β1 sensitive promoter activity reporter assay and co-immunoprecipitated with Smad3. (A) HBECs were transfected with a TGF-β1/Smad-responsive reporter 3TP-luciferase (3TP-Luc) and increasing amounts of pCIG empty vector or pCIG-Sox2 (0.25 µg and 0.5 µg) in the presence or absence of TGF-β1 (2 ng/ml). Sox2 inhibits TGF-β1-mediated reporter activation. (B) Coimmunoprecipitations performed on lysates from HBECs expressing Sox2-V5 and FLAG-Smad3 demonstrated an interaction between Sox2 and Smad3. (C) Sox2 inhibits Smad3-induced activation of 3TP-Luc in the presence or absence of TGF-β1 in HBECS. IP, immunoprecipitation; IB, immunoblot; IgG, immunoglobulin negative control. Graphs depict representative results from three experiments±standard deviation of the mean.

Since the loss of *Sox2* resulted in decreased bronchiolar cell proliferation, we sought to identify a mechanism by which Sox2 influenced cell proliferation. Sox17, another member of the Sox family of transcription factors, promotes proliferation, in part by direct binding to Smad3, thus blocking TGF-β induced cell cycle repression [33]. Since TGF-β/Smad signaling is a major anti-proliferative pathway in multiple epithelial cell types, and is active in the postnatal mouse bronchiolar epithelium [34], we tested the effect of Sox2 on TGF-β1/Smad3 mediated gene transcription. Sox2 inhibited TGF-β1 and Smad3 induced activation of a 3TP luciferase reporter construct in HBECs (Fig. 7A,C). Coimmunoprecipitation assays demonstrated that Sox2 forms a complex with Smad3 (Fig. 7B). Taken together, these data provide a potential mechanism by which loss of Sox2 may influence cell proliferation.

## Discussion

Deletion of *Sox2* from Clara cells in the postnatal bronchiolar epithelium caused the progressive loss of ciliated, Clara, and goblet cells and an inability to produce goblet cells in response to allergen challenge. Taken together, expression of Sox2 in Clara cells is required for differentiation and/or maintenance of ciliated, Clara, and goblet cells in bronchiolar epithelium after birth. The findings support the concept that Clara cells serve as the common progenitors of ciliated, Clara, and goblet cells in a process requiring *Sox2*. Remarkably, *Sox2* ^Δ/Δ^ mice survive normally in the vivarium in spite of the widespread loss of ciliated, Clara, and goblet cells from conducting airways.

**Figure 8:**
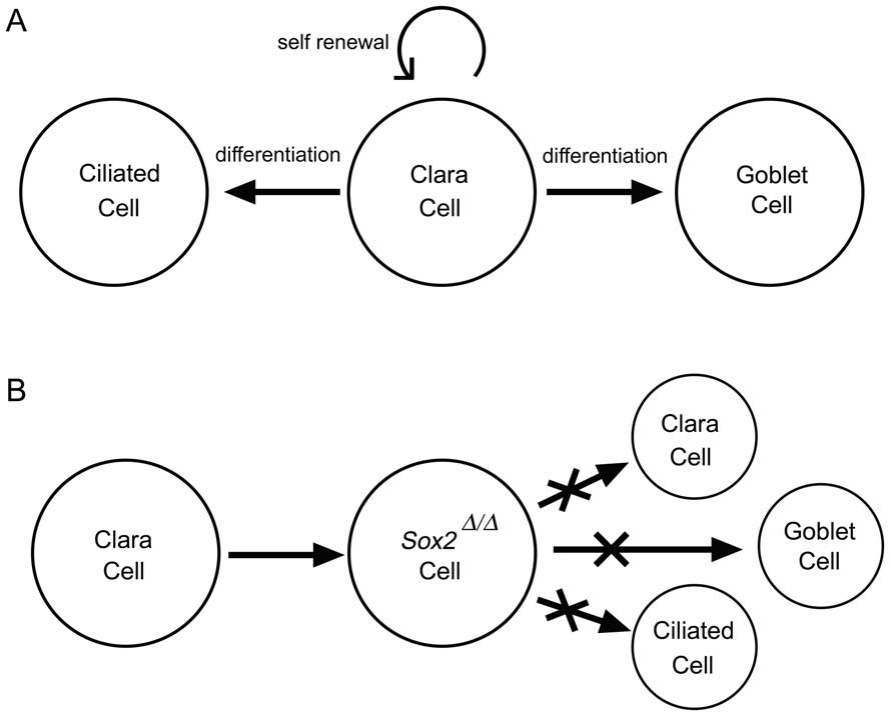
Model in which *Sox2* is required for Clara cell self renewal and differentiation of facultative progenitors of the bronchiolar epithelium. (A) The Clara cell is postulated to be the facultative progenitor cell of the bronchiolar epithelium, possessing differentiated cell characteristics yet capable of self-renewal and differentiation into ciliated or goblet cells. (B) Loss of *Sox2* from Clara cells results in an epithelium lacking differentiated cells, consistent with the Clara/facultative stem cell model. Sox2 is required for normal proliferation during perinatal growth and for the postnatal differentiation of Clara, ciliated, and goblet cells in the bronchiolar epithelium.

The Clara cell of the intrapulmonary airway is capable of self-renewal and transdifferentiation into ciliated and goblet cells [16], [17], [18], and has been termed a facultative progenitor in lung homeostasis [35]. In the present study, we utilized the rat *Scgb1a1* promoter that is specifically active in Clara but not in ciliated or neuroepithelial cells. The loss of Clara, ciliated and goblet cells in response to *Sox2* deletion are consistent with the proposed role of Clara cells as progenitor cells in bronchioles, and show that *Sox2* is necessary for normal proliferation and differentiation of the major epithelial cell types lining the bronchioles (Fig. 8).

Experiments observing the steady-state pattern of chimeric labeled mouse embryonic stem cells [36], and pulse-lineage-labeled newborn Clara cells [17] show that for homeostasis of the intrapulmonary respiratory epithelium, most progenitor cells (e.g. Clara cells) are randomly distributed throughout the bronchioles and are not associated with particular anatomical niches. Interestingly, unlike bronchiolar Clara cells, numbers of tracheal Clara cells that were labeled with a conditional Cre-ER in the newborn period, steadily declined as the mice aged. Thus, progenitor cell activity in the trachea may be somewhat different than that in the bronchioles [17], findings consistent with previous studies demonstrating the importance of tracheal basal cells as progenitors during repair [5], [37], [38]. Epithelial cells in the submucosal glands and basal cells in larger airways are able to contribute to epithelial repair. Although recent studies demonstrate that progenitor cells in rare anatomical niches were not responsible for bronchiolar maintenance, there is evidence that they play a role in lung repair after extensive injury [36], [37], [38]. Bronchio-alveolar stem cells (BASCs) have been proposed as a uniquely proliferative stem cell [7], [8], although recent evidence supports the concept that Clara cells or toxicant resistant Clara cells are the primary progenitors during repair of the postnatal lung airway [36], [39].

The present study and recent findings, in which *Sox2* was deleted in the embryonic lung and trachea, support the concept that progenitor cell relationships are somewhat distinct in the proximal and peripheral airways. An *Nkx2.5-Cre* construct was used recently to delete *Sox2* from the ventral epithelial domain of the anterior foregut prior to lung budding and the consequences on tracheal development were examined [19]. The deletion of *Sox2* using *Nkx2.5* occurred earlier than in the present model and resulted in abnormal laryngeal/tracheal cartilage formation and perinatal death. In that study, a small subset of tracheal cells lacked markers of differentiation and numbers of tracheal Clara and ciliated cells were reduced. In contrast to findings in the present study, increased numbers of tracheal goblet cells were observed and tracheal proliferation was normal following the early deletion of *Sox2* from the embryo [19]. In the present study, *Sox2* was deleted in the perinatal/postnatal period, after lung branching morphogenesis was complete, and its consequences on postnatal growth and allergen challenge were examined. Perinatal deletion of *Sox2* resulted in progressive loss of both ciliated and Clara cells in the bronchiolar epithelium. In response to the allergen, goblet cell differentiation was absent in the *Scgb1a1-Cre Sox2* ^Δ/Δ^ mice in spite of extensive inflammation. Goblet cells were absent at baseline and after allergen challenge as indicated by mucin, Muc5AC, and Spdef staining in the bronchioles of the *Scgb1a1-Cre Sox2* ^Δ/Δ^ mice. The significant temporal and spatial differences in which *Sox2* was deleted are likely responsible for the differences in goblet cell differentiation seen in these two systems.

A tamoxifen-inducible *CMV-CreER* construct was used recently to delete *Sox2* in adult mice and its effect on tracheal homeostasis studied [19]. In the adult tracheal epithelium, reduced cell proliferation and cell height and increased cell width were observed. In the trachea, numbers of Clara cells were not reduced after deletion of *Sox2*, perhaps indicating the importance of basal cells to the homeostasis of pseudostratified epithelium of the proximal airway. Recent lineage analysis of Clara cells in the trachea and bronchioles support their role in bronchiolar homeostasis, but not in the trachea where other cell types, presumably basal cells, play an important role.

Clara cells are a source of bronchiolar ciliated cells after birth [16], [17], and ciliated cells are long-lived [40]. In the present mouse model, the progressive loss of Clara cells occured during the rapid growth phase (0–4 wks) and was faster than loss of ciliated cells, as indicated by the expression of FoxJ1 and α-tubulin. The lack of cleaved Caspase 3 staining after deletion of *Sox2* suggests that the loss of cell number is not related to apoptosis. Thus it is likely that the progressive loss of the columnar morphology is mediated by the decreased proliferation, the gradual loss of ciliated cells that were differentiated before the deletion of *Sox2* in their progenitors (Clara cells) and the rapid loss of differentiated features of Clara cells after deletion of *Sox2*. These findings suggest that Sox2 plays a role in Clara cell differentiation, proliferation and progenitor cell capacity.

The deletion of *Sox2* from the bronchial epithelium did not influence the expression of Nkx2.1 or Foxa2, transcription factors important for the expression of differentiation markers. Thus the loss of the Clara cell marker Scgb1a1 (a direct target of both Nkx2.1 and Foxa2) is not caused by the loss of Nkx2.1 or FoxA2. In the present study, *in vitro* promoter assays demonstrated that Sox2 was not a synergistic partner of Nkx2.1 or FoxA2 in the activation of *Scgb1a1*. The ubiquitous presence of Sox2 in normal Clara, ciliated, and goblet cells, and the activation of the *Scgb1a1*, *FoxJ1*, and *Agr2* promoter-luciferase constructs by Sox2 support the role of Sox2 as a positive regulator of these differentiation markers. Taken together, the results demonstrate that Sox2 is necessary for differentiation and maintenance of Clara, ciliated, and goblet cells from Clara cell progenitors, and may play a role in directly regulating the expression of differentiation dependent genes in each of these cell types. Recent studies demonstrated the loss of both Nkx2.1 and FoxA2 during allergen induced differentiation of goblet cells [18]. The lack of goblet cells during allergen challenge to *Sox2^Δ/Δ^* mice supports the concept that Clara cell differentiation, dependent upon Sox2, is required for the differentiation of Clara cells into goblet cells following allergen exposure. The bronchiolar epithelium of the *Sox2^Δ/Δ^* mice lacked both Muc5AC and Spdef (Fig. 4E–H). Perhaps consistent with the requirement for Sox2 in goblet cell differentiation, Sox2 activated a *Muc5AC*-luciferase reporter in colorectal tumors [41], and *Muc5B* mRNA was increased in lung when Sox2 was overexpressed in a transgenic mouse model [25].

In the present study, cell proliferation was significantly reduced in the bronchioles after perinatal deletion of *Sox2* using a *Scgb1a1-Cre* transgene, a finding consistent with reduced cell proliferation seen in the adult trachea after deletion of *Sox2* from mature trachea using an *CMV-CreER* transgene [19]. While mechanisms controlling its role in bronchiolar cell proliferation are unclear at present, Sox2 promotes proliferation in breast cancer cell lines [42] and the loss of *Sox2* promotes terminal differentiation of neuronal stem cells [43]. The functions of Sox2 are highly dependent on cell types and developmental cassettes. Sox2 mediates cellular reprogramming required for pluripotent stem cell activity in fibroblasts. Sox2 interacts with multiple transcriptional partners and co-activators that are influenced by developmental, cellular, and tissue dependent factors. TGF-β/Smad3 signaling is a potent anti-proliferative pathway in multiple epithelial types, including respiratory epithelial cells, and promotes squamous differentiation of bronchiolar epithelial cells *in vitro* [44]. In the present study, we observed that Sox2, like Sox17, bound to Smad3 and inhibited its activity, providing a potential mechanism by which Sox2 may influence cell proliferation and differentiation.

### Summary

In summary, the expression of Sox2 in Clara cells of the bronchiolar epithelium was required for its normal differentiation and proliferation after birth. The present data demonstrate the importance of both Sox2 and Clara cells (the latter serving as facultative progenitors of Clara, ciliated, and goblet cells) in proximal airway differentiation and homeostasis. Sox2 was required for the expression of Spdef, a gene required for postnatal goblet cell differentiation and mucus hyperproduction in response to pulmonary allergen sensitization. In spite of the extensive loss of Clara, ciliated, and goblet cells, mice in which *Sox2* was extensively deleted from the respiratory epithelium do not develop spontaneous infections and survive normally in the vivarium, indicating an unexpected compensatory capacity in the maintenance of homeostasis in spite of extensive changes to the structure of the conducting airway.

## Materials and Methods

### Transgenic Animals

The *Sox2* ^floxed^ allele was generated in an SV129 background by flanking the single exon of the endogenous *Sox2* allele with loxP sites via homologous recombination [45]. The *Scgb1a1-Cre* transgenic mouse [46] was kindly provided by Dr. Steven Shapiro (Pittsburg, PA) and maintained on a SV129/C57Bl6 background. Mice were crossed to produce *Scgb1a1Cre/Sox2^flox/flox^* mutants with *Scgb1a1-Cre*, and *Sox2^flox/flox^* control littermates. No morphologic or histologic differences were observed between the *Scgb1a1-Cre* and *Sox2^flox/flox^* mice. Mice of all genotypes survived normally after birth and up to 6 months of age in our vivarium.

### Animal Husbandry

Mice were maintained in a pathogen-free environment in accordance with protocols approved by the Institutional Animal Care and Use Committee of the Cincinnati Children's Hospital Research Foundation. Food and water were provided *ad libitum* in temperature-controlled isolation cages on a 14 hr light/10 h dark cycle. There was no serological evidence of pulmonary pathogens or bacterial infections in sentinel mice maintained within the colony.

### Tissue Preparation and Immunostaining

Tissue harvesting was preceded by administration of 0.1 mL anesthetic (ketamine, xylazine, and acepromazine) and then exsanguination by severing the inferior vena cava. Mouse lungs obtained on postnatal day 5 and thereafter were inflation fixed by gravity with 4% PFA (paraformaldehyde in phosphate buffered saline (PBS)(20 mM Tris·HCl, pH 7.6, 137 mM NaCl)) at 25 cm hydrostatic pressure for 1 minute. Embryonic and adult lungs were immersed overnight in 4% PFA at 4°C, washed in PBS followed by dehydration in a series of ethanol solutions prior to paraffin embedding. Paraffin sections were melted in a 60°C oven and deparaffinized in xylene, followed by rehydration in ethanol washes. Peroxidase treatment in methanol with 0.5% hydrogen peroxide was followed by heat-assisted antigen retrieval in 0.01 M sodium citrate buffer (pH 6.0). Blocking was performed for 2 hrs at room temperature using 4% goat or 4% donkey serum followed by overnight incubation with primary antibody at 4°C. Antibodies used were Sox2 (WRAB-Sox2, 1∶2000, Seven Hills Bioreagents, Cincinnati, OH), Scgb1a1 (WRAB-Ccsp, 1∶4000, Seven Hills Bioreagents), Nkx2.1 (WRAB-Ttf1, 1∶2000, Seven Hills Bioreagents), FoxA2 (WRAB-FoxA2, 1∶5000, Seven Hills Bioreagents), FoxJ1 (generated internally, 1∶6000), acetylated α-tubulin (catalog T7451 Clone6-11B-1, IgG2b, 1∶2000, Sigma, St Louis, MO), Keratin-14 (Clone LL002, IgG3, 1∶100, Neomarkers, Fremont, CA), p63 (sc-8344, 1∶200, Santa Cruz Biotechnology Inc., Santa Cruz, CA), Ki-67 (m7249, 1∶500 Dako, Carpintera, CA), CGRP (catalog 8198, 1∶3000, Sigma), and cleaved Caspase 3 (1∶1000, rabbit polyclonal; R&D Systems, Inc., Minneapolis, MN). Rinsed sections were incubated with biotinylated secondary antibodies directed to primary antibody host IgGs (7.5 ug/ml; Vector Laboratories, Burlingame CA), visualized with the Vectastain Elite ABC kit (Vector Laboratories, Burlingame, CA) using nickel-diaminobenzidine as a substrate, enhanced with Tris-cobalt, and counterstained with 0.1% nuclear-fast red. Rabbit polyclonal antibody was generated against a fragment of synthetic mouse Sox2 protein (aa 111–140) conjugated to keyhole limpet hemocyanin. Peptide synthesis and conjugation were performed by AnaSpec Inc. (San Jose, CA). Immunohistofluorescence was performed on 5-µm-thick sections using antibodies generated to Sox2 (1∶200), CCSP (1∶2000, goat polyclonal, sc-9772), acetylated α-tubulin (1∶2000), Ki-67 (1∶100), and secondary antibodies conjugated with Alexa Fluor 594, Alexa Fluor 488 fluorochromes (1∶200, Molecular Probes, Invitrogen Corp., Carlsbad, CA). The slides were examined by microscopy using dual fluorescent labeling and a Zeiss Axioplan 2 Imaging Universal Microscope with an Axiocam MRm black and white digital camera (Axiovision Release 4.3).

### Pulmonary Allergen Exposure

At approximately 6 weeks of age, *Sox2^Δ/Δ^* (n = 6) and control (n = 5) mice were sensitized by intraperitoneal injection of 0.1 mg ovalbumin (OVA, Sigma, grade V) and 1 mg aluminum hydroxide adjuvant (alum) on days 0 and 14. Intranasal treatment with 50 µg OVA was performed on days 25 and 28 as previously described [47]. Lungs were harvested on day 30.

### Cell Proliferation and Density

Immunofluorescence staining for Sox2 and for the proliferation marker Ki-67 was performed on lung sections from 4 week old *Sox2^Δ/Δ^* (n = 3) and control (n = 3) mice. At least 8–12 images containing a total of 200 cells and approximately 1400–1500 um of bronchiolar epithelium, were examined at a magnification of 40X for each of the *Sox2* ^Δ/Δ^ mice; and 7–8 images, containing 200 cells and approximately 1200–1300 um of bronchiolar epithelium were examined for each of the control mice. Total cell counts, number of Ki-67 positive cells, and length of bronchiolar epithelium examined were obtained from contiguous Sox2-negative cells for the *Sox2* ^Δ/Δ^ mice. Cell counts and bronchiolar epithelial lengths were determined using MetaMorph® imaging software (Molecular Devices, Downingtown, PA). Cell width, height, and density were calculated from the raw data measurements. Statistical differences were assessed by the Student's *t* test when data were normally distributed or by the Mann-Whitney Rank Sum test when data did not meet equal variance or normal distribution criteria.

### Plasmids

The pCIG-Sox2 (mouse cDNA) plasmid and pcDna6.1-Sox2-V5 epitope-tagged (mouse cDNA) construct were kind gifts from Dr. Aaron Zorn and Dr. James Wells [48]. The 3TP-luciferase (3TP-Luc) TGF-β/Smad reporter plasmid was obtained from Dr. Jeff Molkentin (Cincinnati Children's Hospital Research Foundation), and the FLAG-Smad3 expression vector (Addgene plasmid 14052) was generated by Dr. Joan Massague (Memorial Sloan-Kettering Cancer Center) [49]. The reporter plasmids, *Scgb1a1-luciferase* (2.3 kb rat *Scgb1a1* promoter-pGL2), *FoxJ1-luciferase* (3.4 kb mouse *FoxJ1* promoter-pGl3), *Agr2-luciferase* (1.6 kb mouse *Agr2* promoter-pGl3), *Spdef-luciferase* (2.8 kb mouse *Spdef* promoter-pGl3), express the *luciferase* gene under control of each promoter. The expression plasmids *pRc/CMV-Nkx2.1* (rat cDNA) and *pRc/CMV-FoxA2* (rat cDNA) have been previously described [50], [51].

### Cell Culture and TGF-β Reporter Assay

Immortalized Human Bronchial Epithelial Cells (HBECs) were maintained as previously described [52]. HBECs were seeded in 6-well culture plates at 1×10^5^ cells per well and transfected using FuGENE6 (Roche). Empty vectors (pCIG or pcDna6.1) and pCMV-β-galactosidase vectors (Clontech) were used to normalize total DNA and transfection efficiency, respectively. Recombinant human TGF-β1 (2 ng/ml; R&D Systems) was added directly to the culture medium where indicated. Cells were harvested 24 h post-transfection and luciferase activity was measured using a Luciferase Assay System kit (Promega) and normalized to β-galactosidase activity. Experiments were performed three times in triplicate and statistical significance was determined using paired Student's *t* test.

### Co-Immunoprecipitation of Smad3 and Sox2

HBECs were seeded in 10 cm plates and transfected at ∼70–80% confluence with Sox2-V5 and FLAG-Smad3 expression vectors using FuGENE6 transfection reagent (Roche). Cells were harvested 24 h post-transfection and lysed for 30 min at 4°C in NETN buffer (20 mM Tris, pH 8.0; 100 mM NaCl; 1 mM EDTA; 0.5% NP-40; 1 mM PMSF; 10 mM beta-glycerophosphate; 10 µl/ml Protease Inhibitor Cocktail, Sigma). Lysates were precleared using Protein A/G PLUS agarose beads (Santa Cruz) for 1–2 h at 4°C and 4% of the precleared lysate volume was kept as input. Immunoprecipitations were performed using equal volumes of the precleared lysate incubated with EZ view Red Anti-FLAG M2 Affinity gel (Sigma) or Protein A/G PLUS agarose beads (Santa Cruz) and rabbit anti-Sox2 (WRAB-Sox2, Seven Hills Bioreagents, 3 µl) or normal rabbit IgG (1.5 µg; Santa Cruz). After several washes with NETN buffer, samples were eluted by boiling in Laemmli sample buffer including β-ME and subjected to SDS-PAGE. Immunoblots were performed using mouse anti-FLAG-HRP (1∶1000; Sigma) and mouse anti-V5-HRP (1∶2500; Invitrogen) antibodies and developed using ECL reagents.
